# Asymmetric organocatalyzed synthesis of coumarin derivatives

**DOI:** 10.3762/bjoc.17.128

**Published:** 2021-08-03

**Authors:** Natália Menezes Moreira, Lorena Suelen Ribeiro Martelli, Arlene Gonçalves Corrêa

**Affiliations:** 1 Centre of Excellence for Research in Sustainable Chemistry, Department of Chemistry, Federal University of São Carlos, 13565-905 São Carlos, SP – Brazil

**Keywords:** asymmetric synthesis, green chemistry, 2*H*-chromen-2-one, organocatalysis

## Abstract

Coumarin derivatives are essential scaffolds in medicinal and synthetic chemistry. Compounds of this class have shown important activities, such as anticancer and antiparasitic, besides the commercially available drugs. These properties led to the development of efficient and greener synthetic methods to achieve the 2*H*-chromen-2-one core. In this context, the advances in asymmetric organocatalyzed synthesis of coumarin derivatives are discussed in this review, according to the mode of activation of the catalyst.

## Introduction

**Figure 1:**
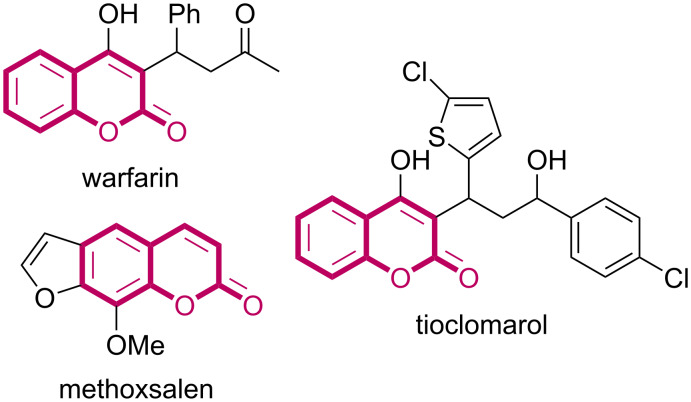
Coumarin-derived commercially available drugs.

Coumarins are important naturally occurring plant constituents and display a wide range of pharmacological and biological activities, such as anticancer [1], antibacterial [2], and antifungal [3]. Moreover, coumarin derivatives have shown activity against neglected diseases as leishmaniasis [4], tuberculosis [5–6] and Chagas’ disease [7]. Examples of coumarin-derived drugs are: methoxsalen, used to treat psoriasis, eczema, vitiligo, and some cutaneous lymphomas; warfarin, an anticoagulant, used to treat blood clots such as deep vein thrombosis and pulmonary embolism, and to prevent stroke; and tioclomarol, also an anticoagulant, that is a long-acting vitamin K antagonist (Figure 1) [8].

**Figure 2:**
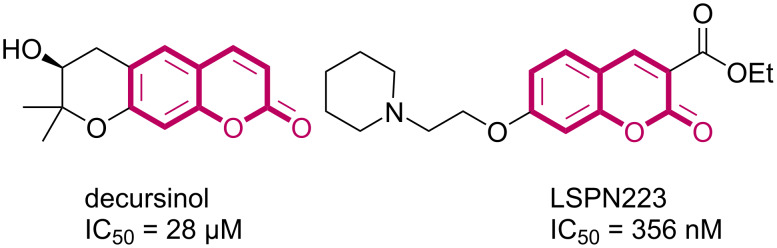
Inhibition of acetylcholinesterase by coumarin derivatives.

This scaffold has also been reported as anti-Alzheimer’s disease [9], such as the natural product decursinol, isolated from *Angelica gigas* [10]. In this sense, our research group has synthesized and evaluated a library of coumarin derivatives as acetylcholinesterase inhibitors [11–13], being LSPN223 the most potent compound (Figure 2).

Furthermore, coumarin derivatives have been used as fluorescent probes, laser dyes, fluorescent chemosensors, light absorbers for solar cells, optical brighteners, and organic light emitting diodes (OLEDs) [14–15].

From a synthetic perspective, coumarin derivatives have received much attention due to their pivotal role in organic synthesis [16–18]. The development of efficient synthetic processes with eco-friendliness and sustainability that avoid the extensive use of toxic and hazardous reagents and solvents, as well as harsh reaction conditions, has become paramount in the field of organic synthesis in recent years [19]. In this sense, Molnar et al. published a review on green chemistry approaches to the synthesis of coumarin derivatives [20] and Chandrakar et al. reviewed the developments of multicomponent synthesis of biologically relevant coumarins in aqueous medium [21].

Catalysis is one of the fundamental pillars of green chemistry [22], and the transition-metal-catalyzed synthesis of coumarins has been reviewed by Sharma et al. [23]. More recently, Kanchana et al. published an account on the palladium-catalyzed cross-coupling reactions of coumarin derivatives [24].

Coumarins are a promising scaffold for design and development of bioactive agents, however it possesses a flat system [25]. One of the attractive benefits of introducing chirality in a drug candidate is that it leads to increased complexity to a specific target, i.e., it gives access to a greater diversity of compounds to be explored [26]. In this work, a compilation of the enantioselective synthesis of coumarin derivatives using asymmetric organocatalysis is presented, highlighting the proposed mechanism pathways for the formation of the stereogenic centers.

## Review

A plethora of highly effective small‐molecule organocatalysts have enriched the field of organic synthesis [27], including chiral proline derivatives, *N*‐heterocyclic carbenes, chiral thioureas and Brønsted acids as well as phase‐transfer catalysts (PTC), such as the quaternary ammonium salts derived from cinchona alkaloids [28]. Therefore, the asymmetric synthesis of coumarin derivatives is herein presented according to the activation mode, i.e., via covalent or non-covalent bonding. Furthermore, the use of bifunctional catalysts and multicatalysis are discussed as well.

### Catalysis via covalent bonding

Organocatalysts made from chiral secondary amines have been widely used in the last years. According to Jørgensen, in general, the carbonyl functionalization employing amine catalysts can be separated in four different types [29]. When aldehydes are employed, both electrophilic and nucleophilic α-functionalizations are possible, whereas with the use of α,β-unsaturated aldehydes the β-position is functionalized with nucleophiles and the γ-position with electrophiles.

**Scheme 1:**
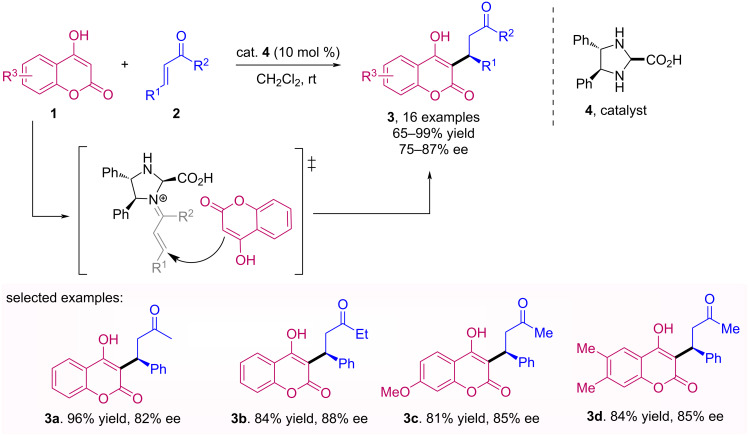
Michael addition of 4-hydroxycoumarins **1** to α,β‐unsaturated enones **2**.

In this sense, Jørgensen and colleagues have developed the first organocatalytic asymmetric Michael addition of cyclic 1,3‐dicarbonyl compounds, including 4-hydroxycoumarins **1**, to α,β‐unsaturated enones **2** (Scheme 1). This versatile Michael reaction afforded (*S*)-warfarin (**3a**) and other Michael adducts **3** in high yields and good enantiomeric excess (ee), using (4*S*,5*S*)-4,5-diphenylimidazolidine-2-carboxylic acid (**4**) as catalyst [30].

**Scheme 2:**
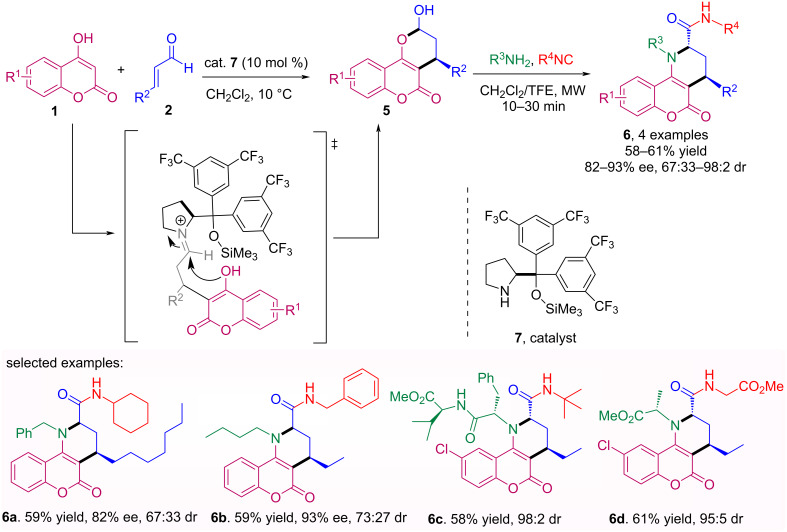
Organocatalytic conjugate addition of 4-hydroxycoumarin **1** to α,β-unsaturated aldehydes **2** followed by an IMCR.

Based on this pioneer work, our research group described an efficient, highly stereoselective, one-pot process comprising an organocatalytic conjugate addition of dimedone or 4-hydroxycoumarin **1** to α,β-unsaturated aldehydes **2** followed by an intramolecular isocyanide-based multicomponent reaction (IMCR) [31]. The enantioenriched hemiacetals **5** were obtained using the Jørgensen catalyst **7** as previously described by Rueping et al. [32]. This approach enables the rapid assembly of complex natural product hybrids **6** including up to four different molecular fragments, such as hydroquinolinone, chromene, piperidine, peptide, lipid, and glycoside moieties (Scheme 2).

**Scheme 3:**
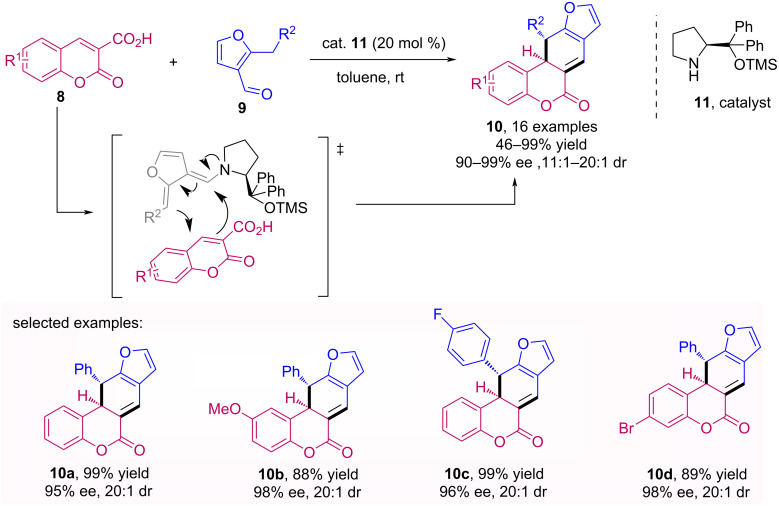
Synthesis of 3,4-dihydrocoumarin derivatives **10** through decarboxylative and dearomatizative cascade reaction.

Bojanowski and co-workers developed a methodology to synthetize 3,4-dihydrocoumarins **10** through a decarboxylative and dearomatizative cascade reaction [33]. This reaction was carried out using coumarin-3-carboxylic acids **8**, 2-alkyl-3-furfural derivatives **9** and diphenylprolinol trimethylsilyl ether **11** as catalyst, and it was possible to obtain 3,4-dihydrocoumarin derivatives with excellent yields, ee and dr (Scheme 3).

**Scheme 4:**
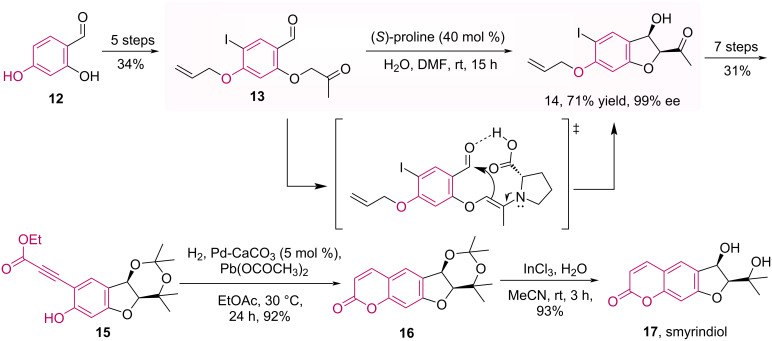
Total synthesis of (+)-smyrindiol (**17**).

Using a completely different strategy from the above discussed, in which the coumarin core was the starting material in the asymmetric organocatalyzed reaction, the Enders group described the use of (*S*)-proline as catalyst in an intramolecular aldol reaction, enabling a new strategy to obtain coumarin natural products [34]. As for example, the total synthesis of (+)-smyrindiol (**17**), a linear dihydrofuranocoumarin isolated from the roots of *Smyrniopsis aucheri*, was developed [35]. The 5-enolexo aldol key step of this synthesis was performed using 40 mol % of (*S*)-proline and the desired product **14** was obtained in good yield (71%), and high diastereo- and enantioselectivities (Scheme 4). Moreover, the natural product **17** was obtained in 15 steps with 6% overall yield.

**Scheme 5:**
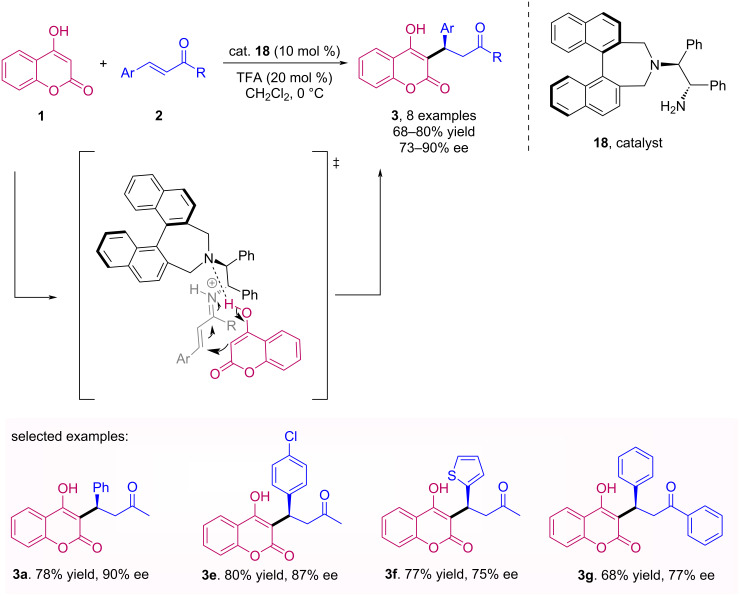
Michael addition of 4-hydroxycoumarin (**1**) to enones **2** through a bifunctional modified binaphthyl organocatalyst **18**.

Although chiral secondary amines have proved to be particularly useful catalysts, primary amines as organocatalysts in asymmetric synthesis have also played a significant role [36]. For instance, Kim et al. described the enantioselective Michael addition of 4-hydroxycoumarin (**1**) by the *Re* face of the enones **2** through a bifunctional modified binaphthyl organocatalyst **18** with primary amine [37]. The reaction occurs through the activation of the enone substrate by formation of an iminium ion intermediate and, in the presence of an acid additive, provides coumarin derivatives **3** with good to excellent yields and moderate to good enantiomeric excesses (Scheme 5). The authors highlighted that the employed organocatalyst **18** is an alternative to those of squaramide and thiourea commonly used with coumarins.

**Scheme 6:**
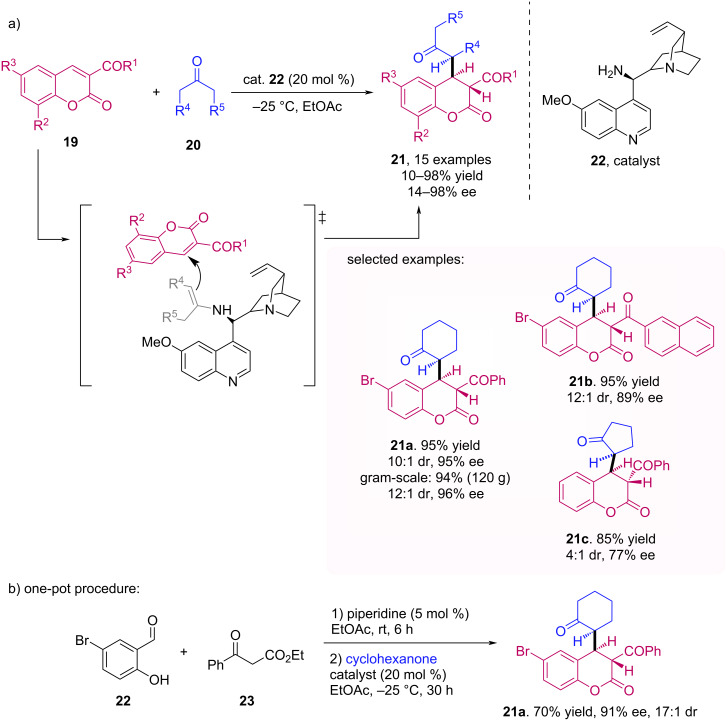
Michael addition of ketones **20** to 3-aroylcoumarins **19** using a cinchona alkaloid-derived primary amine catalyst **22**.

In 2013, Lee et al. reported the enantioselective Michael addition of ketones **20** to 3-aroylcoumarins **19** [38]. For this transformation, the authors used a cinchona alkaloid-derived primary amine catalyst **22** (Scheme 6a). The study was performed with cyclic and acyclic ketones **20** and various 3-aroylcoumarins **19** and the desired products **21** were obtained with good to excellent yields and enantiomeric excesses. Besides, the one-pot synthesis of coumarins followed by the Michael addition step was proven to be a good alternative, affording the desired product with excellent yield and ee. The applicability of the methodology was also demonstrated by a gram-scale experiment, affording the desired product **21a** with excellent yield and ee (Scheme 6b).

**Scheme 7:**
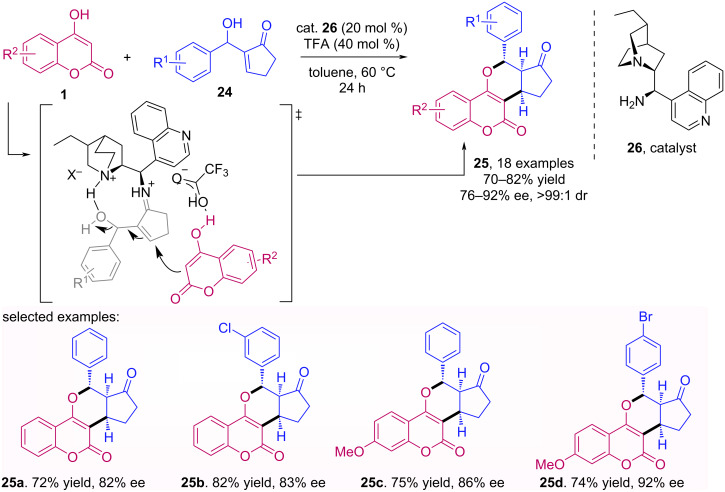
Enantioselective reaction of cyclopent-2-enone-derived MBH alcohols **24** with 4-hydroxycoumarins **1**.

Ren et al. reported an enantioselective reaction of cyclopent-2-enone-derived Morita–Baylis–Hillman (MBH) alcohols **24** with 4-hydroxycoumarins **1** catalyzed by a chiral primary amine derived from dihydrocinchonine **26** in combination with trifluoracetic acid (TFA) as Brønsted acid [39]. The reaction provides pyranocoumarins **25** with three vicinal stereogenic centers in high regio-, diastereo- and enantioselectivities through a tandem allylic alkylation/intramolecular oxa-Michael addition (Scheme 7).

**Scheme 8:**
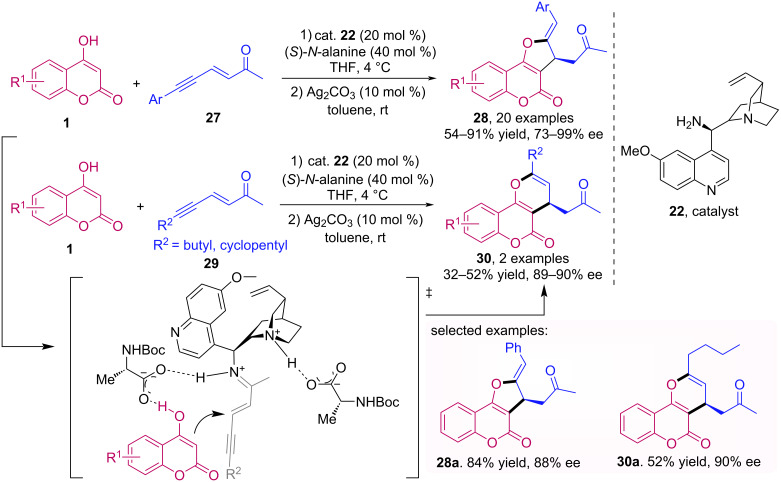
Sequential Michael addition/hydroalkoxylation one-pot approach to annulated coumarins **28** and **30**.

A stereoselective one-pot procedure for the synthesis of five-membered annulated coumarins **28** was described by the group of Enders [40]. Using dual catalysis, with a cinchona primary amine derivative **22** and silver carbonate, a series of functionalized coumarin derivatives **28** were obtained in good yields (up to 91%) and good to excellent enantioselectivities (up to 99% ee) via a Michael addition/hydroalkoxylation reaction (Scheme 8). Interestingly, when alkyl substituted substrates **29** were employed, the corresponding six-membered annulated coumarins **30** were obtained.

**Scheme 9:**
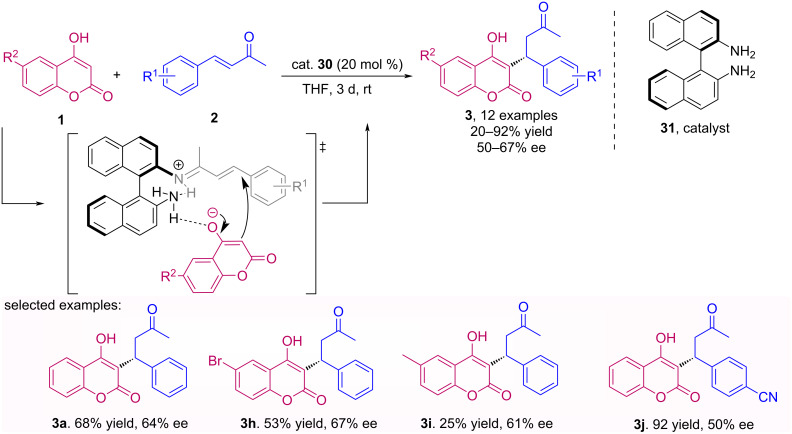
Michael addition of 4-hydroxycoumarins **1** to enones **2** using a binaphthyl diamine catalyst **31**.

The synthesis of (*R)*-warfarin (**3a**) was described by Herrera et al. for the first time using primary aromatic diamines **31** as organocatalysts. The application of this class of catalysts for the Michael asymmetric addition of 4-hydroxycoumarins **1** to enones **2** is interesting from the point of view of organocatalysis, since the presence of two primary amines enables both the formation of an imine ion with the enone and activation of the hydroxycoumarin by hydrogen bonding [41]. Despite the long reaction time (3 days), the desired products **3** were obtained with good to excellent yields and moderate enantiomeric excesses (Scheme 9).

**Scheme 10:**
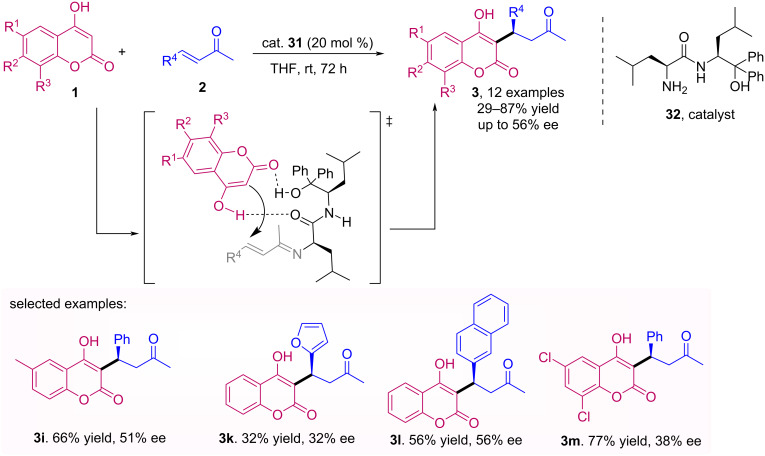
Asymmetric Michael addition of 4-hydroxycoumarin **1** with α,β-unsaturated ketones **2** catalyzed by a chiral primary amino amide **32**.

A new organocatalyst was synthesized by Kumagai et al. and applied in the Michael addition of 4-hydroxycoumarin **1** with α,β-unsaturated ketones **2** [42]. This chiral primary amino amide organocatalyst **32** afforded the desired products **3**, including warfarin (**3a**) in 86% yield, although in moderate enantioselectivity (up to 56% ee) (Scheme 10).

**Scheme 11:**
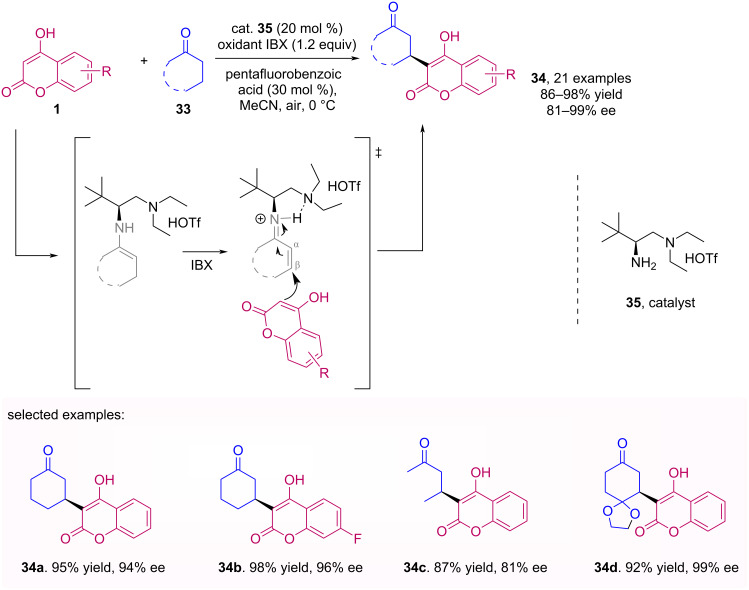
Catalytic asymmetric β-C–H functionalization of ketones via enamine oxidation.

A catalytic asymmetric β-C−H functionalization of ketones **33** with 4-hydroxycoumarins **1** was developed by Zhu et al. [43]. The enamine, formed via reaction of the aminocatalyst **35** with the ketone, is oxidased by IBX resulting in the electrophilic imine, which in turn undergoes a nucleophilic addition of the hydroxycoumarin. The procedure allowed obtaining products **34** with excellent yields and enantiomeric excesses (Scheme 11).

**Scheme 12:**
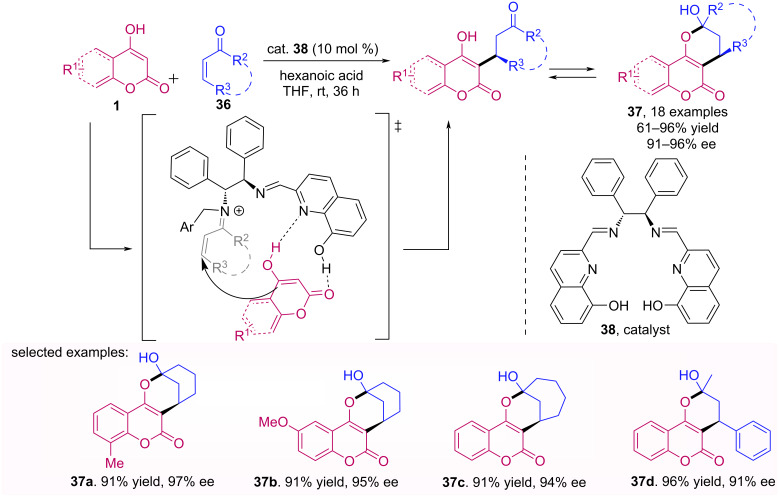
Enantioselective synthesis of polycyclic coumarin derivatives **37** catalyzed by an primary amine-imine catalyst **38**.

Zhu et al. described the asymmetric Michael addition of substituted 4-hydroxycoumarins (**1**) to cyclic enones **36**, using an in situ formed organocatalyst [44]. The proposed transition state includes activations of the enone via an iminium ion and the coumarin by hydrogen bonding. A series of optically active polycyclic pyranocoumarin derivatives **37** was obtained in high yields with excellent enantioselectivities (up to 97% ee) (Scheme 12).

**Scheme 13:**
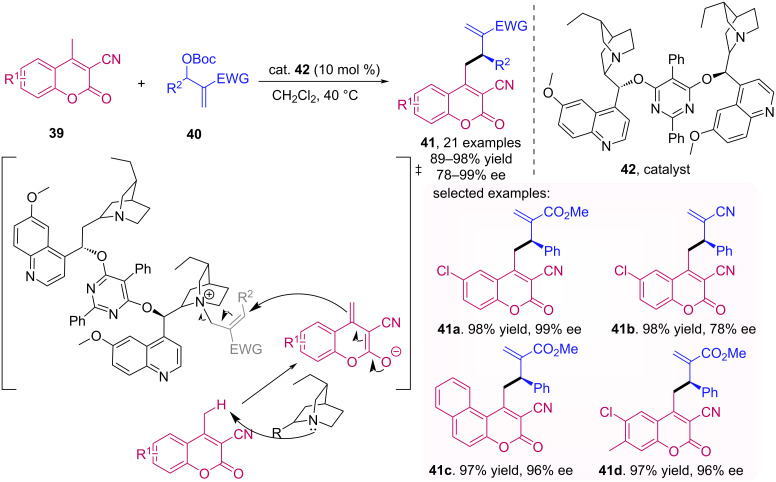
Allylic alkylation reaction between 3-cyano-4-methylcoumarins **39** and MBH carbonates **40**.

Kowalczyk and Albrecht described an allylic alkylation reaction between 3-cyano-4-methylcoumarins **39** and Morita–Baylis–Hillman (MBH) carbonates **40** [45]. In this case, the catalyst (DHQ)_2_PYR **42** activates the MBH substrate and generates the dienolate in the vinylogous coumarin moiety, acting as a base. After the nucleophilic substitution reaction between the coumarin and the activated MBH substrate, it is possible to obtain functionalized coumarins **41** (Scheme 13). Furthermore, the absolute configuration of the stereogenic center was determined by X-ray crystallography.

**Scheme 14:**
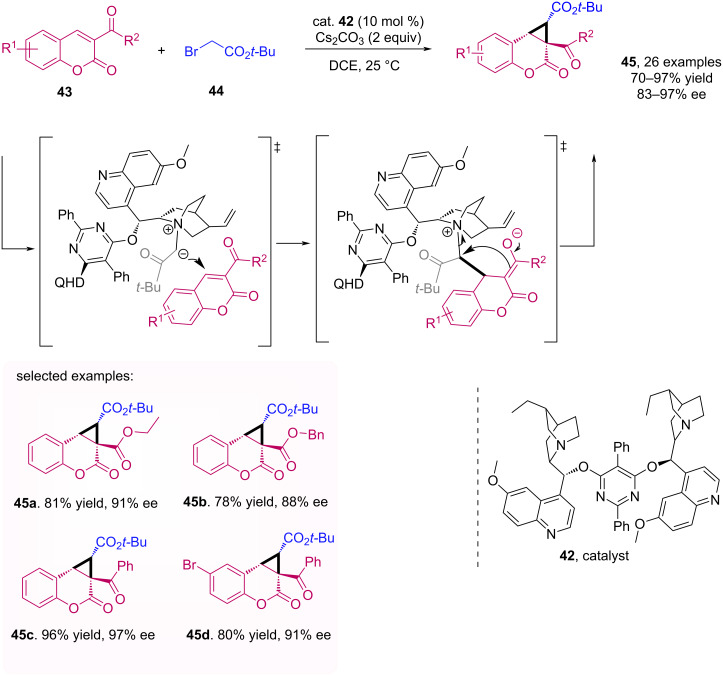
Enantioselective synthesis of cyclopropa[*c*]coumarins **45**.

The enantioselective synthesis of cyclopropa[*c*]coumarins **45** was described by Sun et al. [46]. In this method, the catalyst (DHQ)_2_PYR **42** reacts with *tert*-butyl 2-bromoacetate, and then an ylide is formed by the base Cs_2_CO_3_. After a conjugated addition of this intermediate to the coumarin **43** followed by nucleophilic substitution, the corresponding cyclopropa[*c*]coumarins are formed with good to excellent yields and enantioselectivities (Scheme 14).

**Scheme 15:**
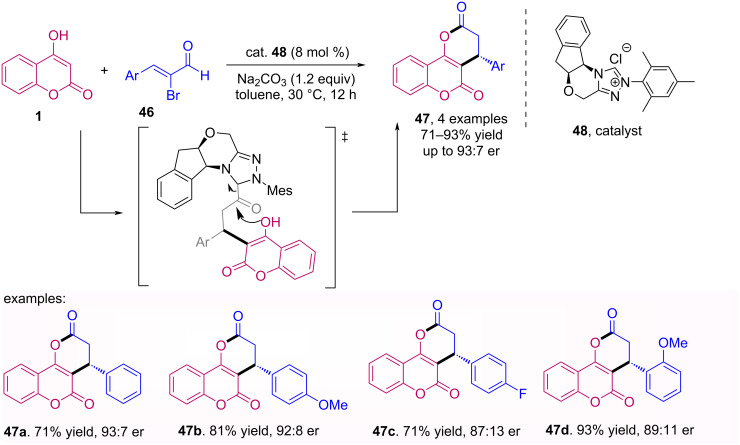
NHC-catalyzed lactonization of 2-bromoenals **46** with 4-hydroxycoumarin (**1**).

*N*-heterocyclic carbenes (NHC) have also been successfully used as organocatalysts, in particular, to obtain coumarin derivatives [47]. In this context, Yetra et al. reported a NHC catalyzed reaction of 2-bromoenals **46** with various heterocyclic C–H acids, resulting in the synthesis of coumarin/quinolinone fused dihydropyranones and dihydropyridinones **47**. The reaction optimization and the scope and limitations study were carried out using an achiral NHC, but the enantioselective version was also performed using 4-hydroxycoumarin (**1**) with the chiral catalyst **48**, as shown in Scheme 15 [48].

**Scheme 16:**
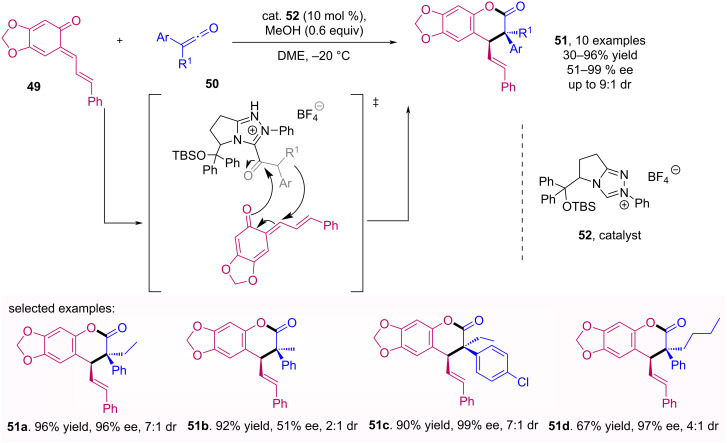
NHC-catalyzed enantioselective synthesis of dihydrocoumarins **51**.

The enantioselective synthesis of dihydrocoumarins **51** from an inverse demand [4 + 2] cycloaddition of ketenes **50** with *o*-quinone methides **49** using carbene catalyst (NHC) **52** was described by Ye and co-workers [49].This transformation resulted in products with moderate to excellent yields and enantiomeric excesses as shown in Scheme 16.

**Scheme 17:**
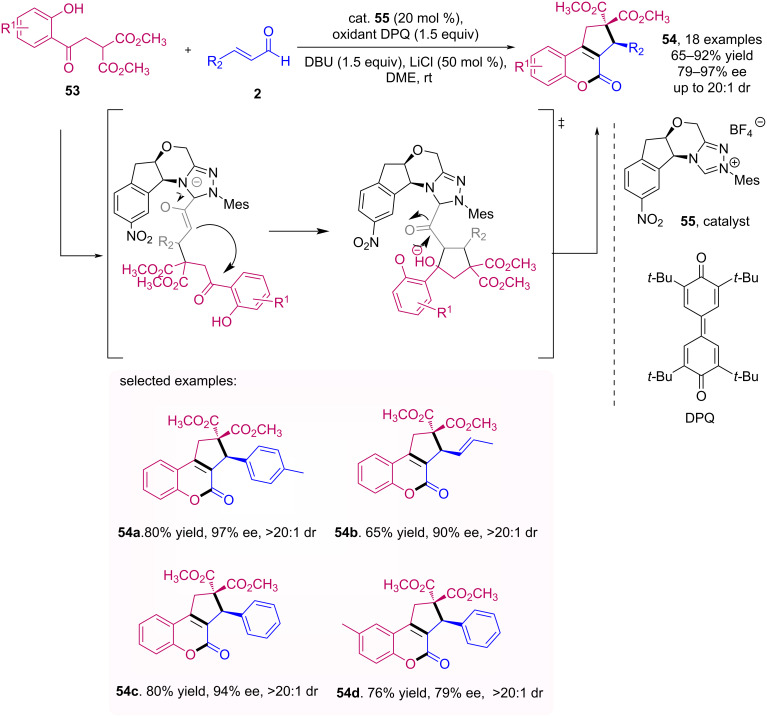
Domino reaction of enals **2** with hydroxylated malonate **53** catalyzed by NHC **55**.

Enders et al. developed the enantioselective synthesis of cyclopenta[*c*]-fused chromenones **54** starting from hydroxylated malonate **53** with enals **2** [50]. The reaction stands out for its good to excellent yields and enantioselectivities when subjected to four sequential reactions mediated by a cooperative catalysis of a NHC organocatalyst with LiCl in the presence of DPQ as an oxidant, as shown in Scheme 17.

**Scheme 18:**
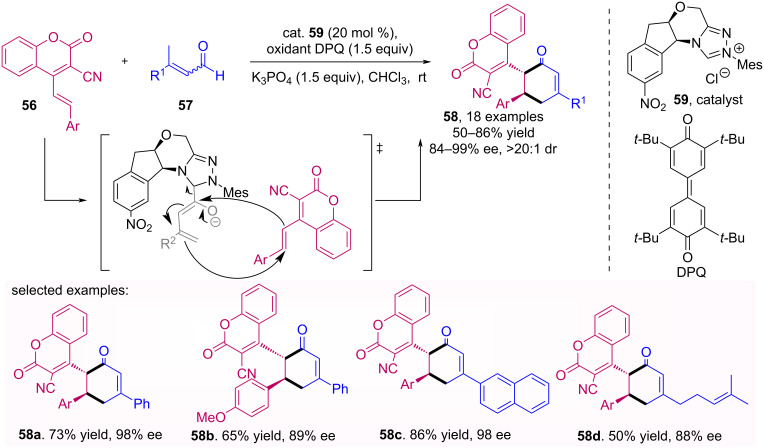
Oxidative [4 + 2] cycloaddition of enals **57** to coumarins **56** catalyzed by NHC **59**.

Recently, Chen et al*.* used a NHC catalyst **59** in γ,δ-difunctionalization of coumarins **56** through an oxidative [4 + 2] cycloaddition with unsaturated aldehydes **57** [51]. The methodology draws attention for the wide variety of products **58** obtained with moderate to excellent yields and enantiomeric excesses (Scheme 18).

### Activation via noncovalent bonding

**Scheme 19:**
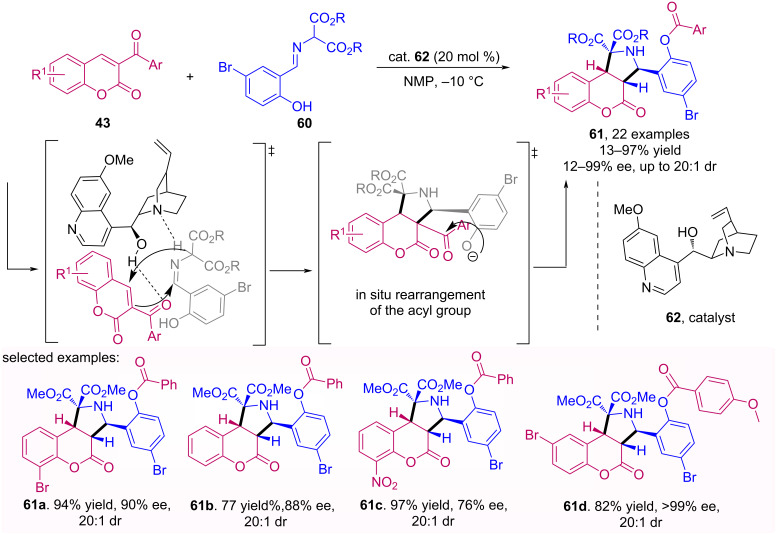
Asymmetric [3 + 2] cycloaddition of coumarins **43** to azomethine ylides **60** organocatalyzed by quinidine **62**.

Besides the activation mode via a covalent bond, as discussed above, the organocatalysts may also proceed by noncovalent activation, in which a hydrogen bond or an ion pair is formed. A broad variety of mono- and bifunctional chiral hydrogen-bonding organocatalysts has been developed, in special using cinchona alkaloid derivatives [52]. In this sense, Lin and colleagues proposed an asymmetric [3 + 2] cycloaddition employing a coumarin dipolarophile **43** with azomethine ylides **60** organocatalyzed by quinidine (**62**) for the formation of fused pyrrolidine compounds through activation of the coumarin substrate by hydrogen bonding [53]. The methodology enabled a high diastereoisomeric control and in most cases with good enantioselectivity of the products. It becomes even more attractive, since it allows an in situ rearrangement of the acyl group that can be used in other functionalization methodologies. However, it presents a limitation relative to the presence of a carbonyl group in the coumarin, since it makes a hydrogen bond with the organocatalyst and when it is replaced by other electron-withdrawing groups, the hydrogen bond formation is blocked, consequently there is no product formation (Scheme 19).

**Scheme 20:**
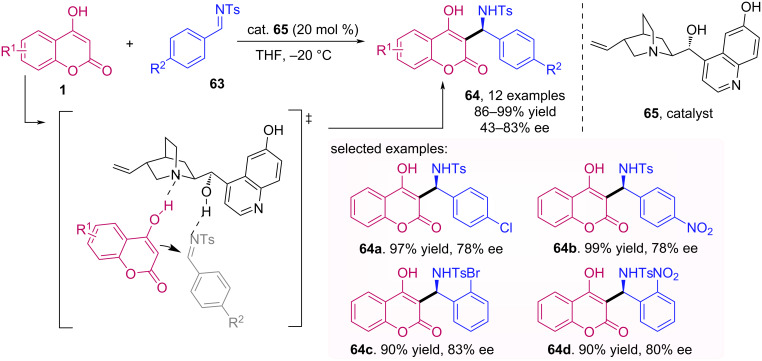
Synthesis of α-benzylaminocoumarins **64** through Mannich reaction between 4-hydroxycoumarins (**1**) and aromatic imines **63** promoted by cupreine (**65**).

Lin et al. described an organocatalyzed Mannich reaction between 4-hydroxycoumarins **1** and aromatic imines **63** for the synthesis of α-benzylaminocoumarins **64** [54]. Among the cinchona alkaloid derivatives evaluated in this reaction, cupreine (**65**) was found to be the best option in terms of yields and enantioselectivities (Scheme 20). Both electron-withdrawing and electron-donating substituents were well tolerated in either coumarin or imine portion, and electron-withdrawing substituents at *ortho*-position of the imine phenyl ring afforded the corresponding products with excellent yields and moderated to good ee.

**Scheme 21:**
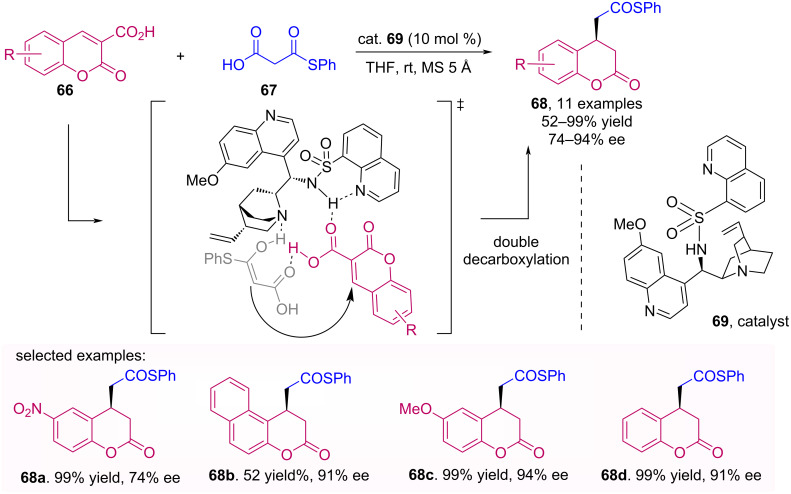
Asymmetric addition of malonic acid half-thioesters **67** to coumarins **66** using the sulphonamide organocatalyst **69**.

The asymmetric addition of malonic acid half-thioesters **67** to coumarins **66** using a sulphonamide organocatalyst **69** was reported by Nakamura et al. [55]. The hydrogen bond between the secondary amine and the coumarin carboxyl provides a nucleophilic addition on the *Re* face, and therefore resulting in products **68** with *R* absolute configuration, with moderate to excellent enantioselectivity followed by two decarboxylations (Scheme 21).

**Scheme 22:**
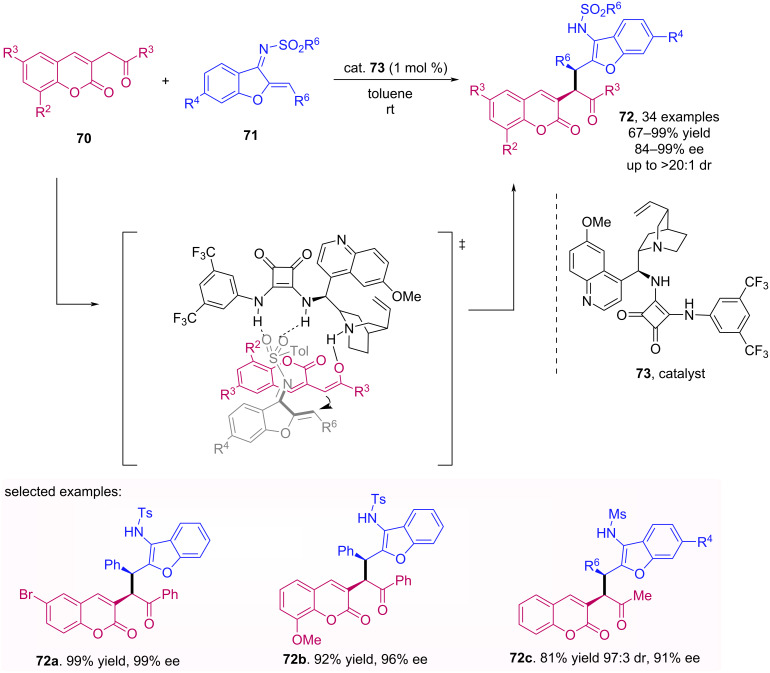
Enantioselective 1,4-addition of azadienes **71** to 3-homoacyl coumarins **70**.

Huang’s group has used azadienes to perform an enantioselective 1,4-addition to afford benzofuran-fused six-membered heterocycles with a squaramide catalyst [56]. Based on their previous work, the authors reported an enantioselective 1,4-addition of azadienes **71** to 3-homoacyl coumarins **70** to achieve benzofuran coumarin derivatives **72** [57]. It was possible to obtain good to excellent diastereo- and enantioselectivities by using a low amount of the catalyst, besides the high yield of the reaction. The best results were obtained using a squaramide cinchona alkaloid catalyst **73** in only 1 mol % loading. In addition, the reaction was also very efficient in a gram-scale experiment, which demonstrates the applicability of the method (Scheme 22).

**Scheme 23:**
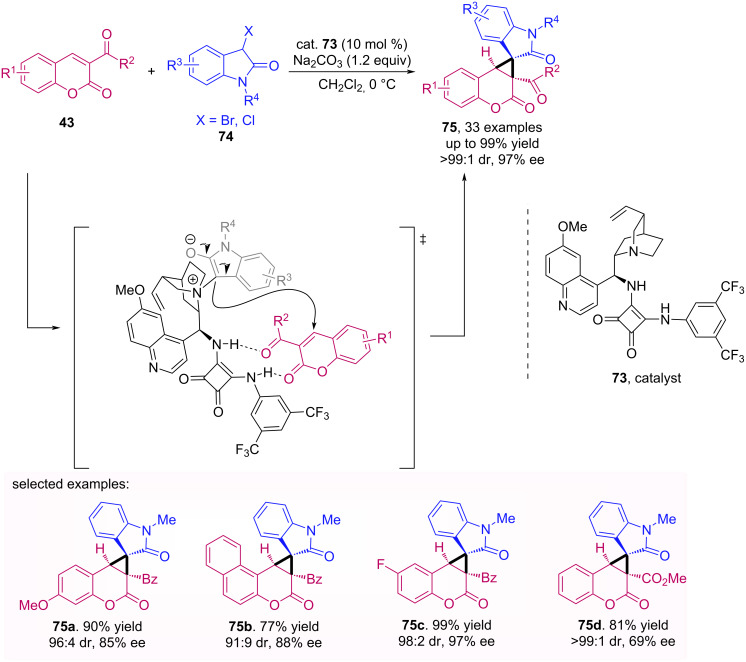
Michael addition/intramolecular cyclization of 3-acylcoumarins **43** to 3-halooxindoles **74**.

More recently, Yuan et al. developed a methodology for the synthesis of spiroonxindole-cyclopropa[*c*]coumarins **75** through the cyclopropanation of 3-acylcoumarins **43** and 3-halooxindoles **74** [58]. The authors chose a quinine-derived squaramide catalyst **73** to perform the [2 + 1] cycloaddition. This catalyst reacts with 3-halooxindole, generating an ammonium salt which is deprotonated by a base, affording an ammonium ylide/enolate. Meanwhile, the *Re*-face attack is favored after interaction of squaramide portion of the catalyst with coumarin. Then, a Michael addition followed by intramolecular cyclization affords the desired product **75**, as shown in Scheme 23.

**Scheme 24:**
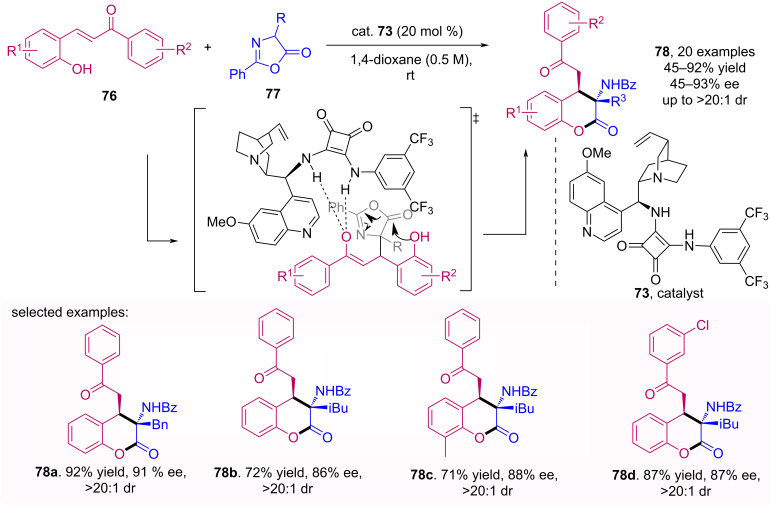
Enantioselective synthesis of 3,4-dihydrocoumarins **78** catalyzed by squaramide **73**.

An enantioselective cascade synthesis of hydrocoumarin **78** mediated by squaramide catalyst with 9-amino-9-deoxy-epi-quinine moiety **73** was reported by Albrecht et al*.* [59]. In this transformation, the authors developed a Michael addition of azlactones to 2-hydroxychalcones **76** followed by the opening of the azlactone **77** ring to form the product of interest, which could be obtained with moderate to excellent yields and enantioselectivities. The protocol used allowed obtaining hydrocoumarins with a wide structural variety and with a diastereoselective control, as shown in Scheme 24.

**Scheme 25:**
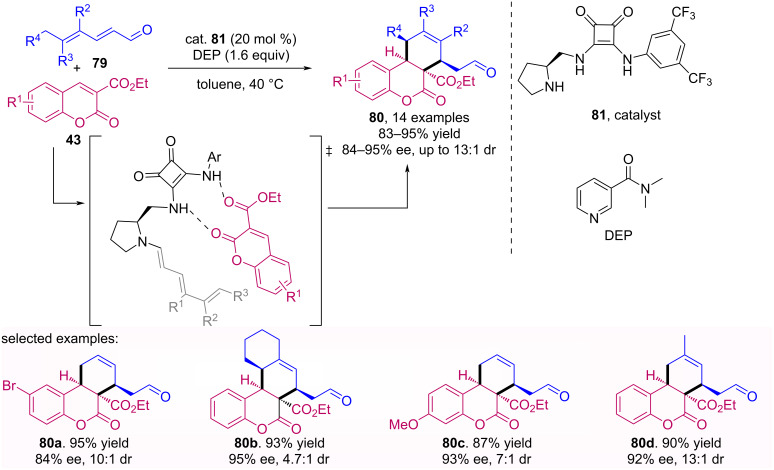
Organocatalyzed [4 + 2] cycloaddition between 2,4-dienals **79** and 3-coumarincarboxylates **43**.

In 2016, Albrecht et al*.* [60] published the synthesis of 3,4-dihydrocoumarins **80** bearing a cyclohexene ring, through [4 + 2] cycloaddition between 2,4-dienals **79** and 3-coumarincarboxylates **43**. This stereoselective transformation was performed using a squaramide **81** derivative catalyst, which activates the aldehyde with the formation of an enamine intermediate and the coumarin through hydrogen bonding, as shown in Scheme 25.

**Scheme 26:**
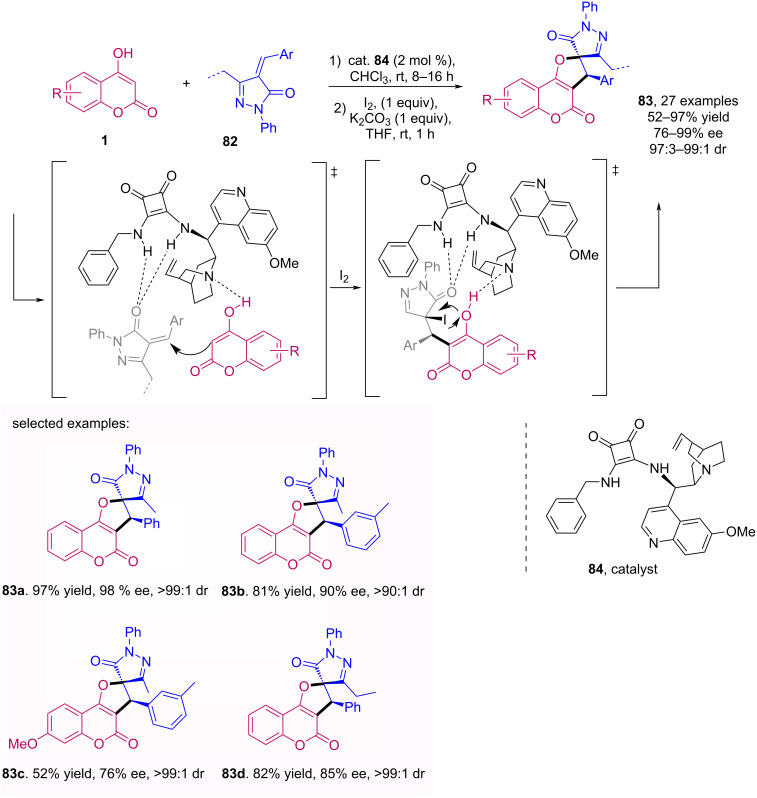
Enantioselective one-pot Michael addition/intramolecular cyclization for the synthesis of spiro[dihydrofurocoumarin/pyrazolone] **83**.

An enantioselective one-pot synthesis of spiro[dihydrofurocoumarin/pyrazolone] **83** mediated by quinine and squaramide catalyst **84** was reported by Xu et al. [61]. The work draws attention for the wide range of compounds obtained with high diastereo- and enantioselectivity and moderate to excellent yields. The authors highlighted that the catalyst also contributes to cyclization, since subjecting the isolated Michael adduct to the second conditions with iodine and K_2_CO_3_ there is a decrease in yield and enantiomeric excess when compared to the one-pot procedure. The obtained products possess a (*R*)-configuration, determined by X-ray crystallography (Scheme 26).

**Scheme 27:**
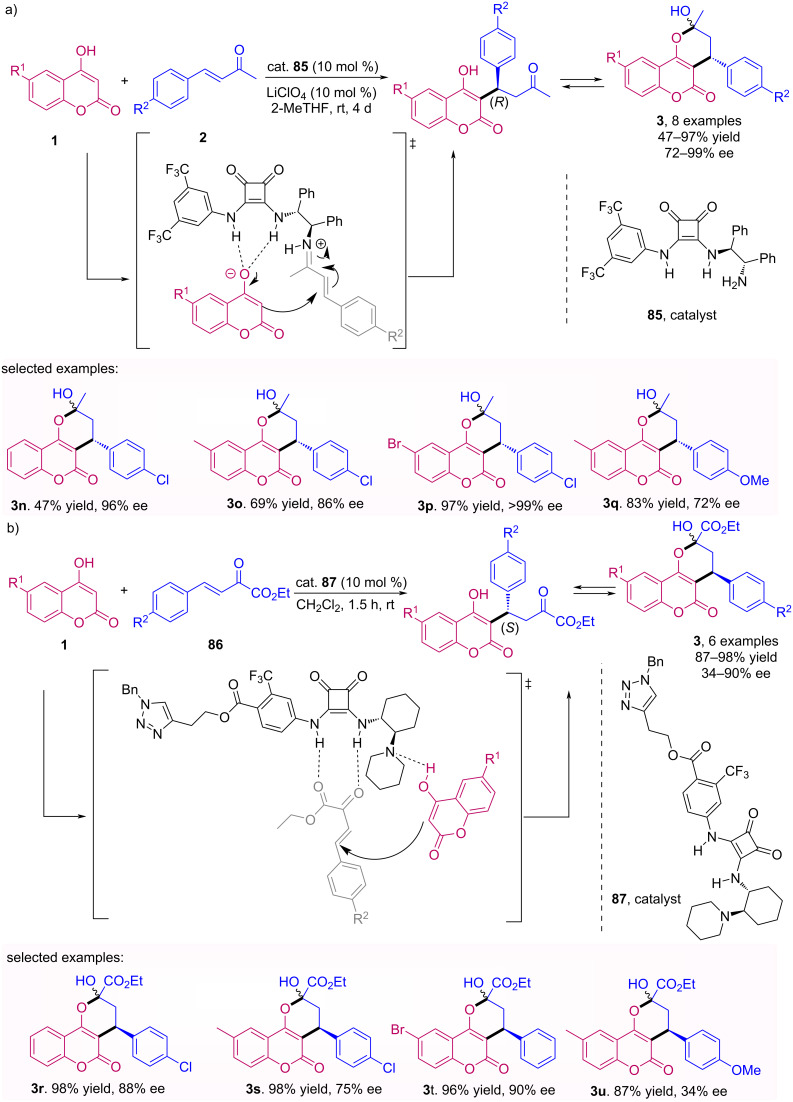
Michael/hemiketalization addition enantioselective of hydroxycoumarins (**1**) to: (a) enones **2** and (b) α-ketoesters **86**.

Sebesta and colleagues described an enantioselective Michael/hemiketalization addition of hydroxycoumarins **1** to enones **2** and ketoesters **86** using squaramide **85** [62]. The methodology developed made it possible to obtain a mixture of open and closed forms of (*R*)-warfarin (**3a**) from a bifunctional catalyst of squaramide by the formation of an iminium ion intermediate with enone and hydrogen bonding with hydroxycoumarin (Scheme 27a). By using the squaramide catalyst with tertiary amine (*S*)-warfarin analogues **3** could be obtained with moderate to excellent enantiomeric excesses (Scheme 27b).

**Scheme 28:**
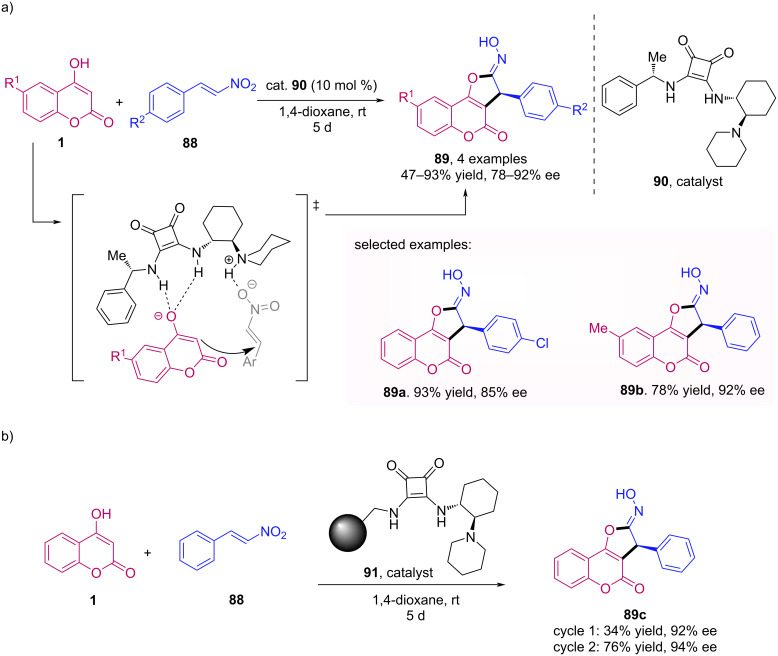
Synthesis of 2,3-dihydrofurocoumarins **89** through Michael addition of 4-hydroxycoumarins **1** to β-nitrostyrenes **88**.

In 2018, Modrocká et al. described the synthesis of 2,3-dihydrofurocoumarins **89** through an enantioselective Michael addition of 4-hydroxycoumarins **1** to β-nitrostyrenes **88**, followed by an intramolecular cyclization [63]. For this transformation, the authors use a squaramide catalyst **90** to perform the enantioselective Michael addition in 1,4-dioxane at room temperature, as shown in Scheme 28a. Moreover, the group tried a reusable immobilised squaramide catalyst **91**, which gave the desired product with high ee in the two first cycles, although the yield of the product in the first cycle was lower (Scheme 28b). Finally, the absolute configuration of the products was determined by ECD analysis.

**Scheme 29:**
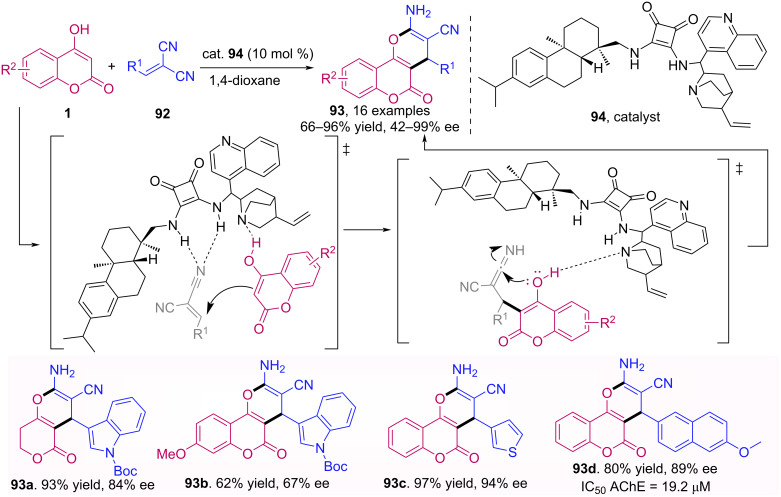
Synthesis of pyrano[3,2-*c*]chromene derivatives **93** via domino reaction between 4-hydroxycoumarins (**1**) and substituted methylene malononitriles **92**.

Zheng et al. described an asymmetric organocatalyzed domino reaction between 4-hydroxycoumarins **1** and substituted methylene malononitriles **92**, affording a variety of pyrano[3,2-*c*]chromene derivatives **93** (Scheme 29) [64]. The catalyst used in this reaction was the dehydroabietylamine-cinchone-squaramide derivative **94**. The products were obtained with good to excellent yields and enantioselectivities with both electron-donating and electron-withdrawing substituents. Additionally, the products were evaluated as acetylcholinesterase (AChE) inhibitors and compound **93d** showed a promising activity.

**Scheme 30:**
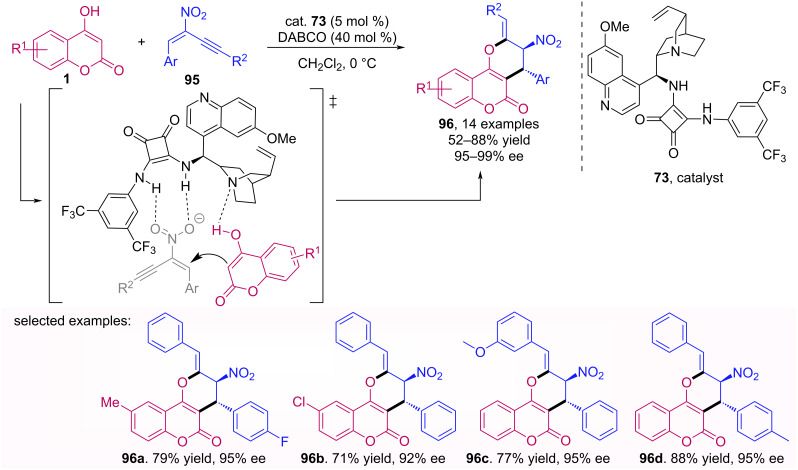
Conjugated addition of 4-hydroxycoumarins **1** to nitroolefins **95**.

Gurubrahaman et al. developed a method for the synthesis of (*Z*)-2-methylenepyrans **96** through a conjugated addition of 4-hydroxycoumarins **1** [65]. This reaction was catalyzed by a bifunctional squaramide **73** and initially both (*Z*)- and (*E*)-isomers were observed, besides the isomer **96** as the major product. After the addition of DABCO, the (*Z*)-isomer became the major product with good to excellent yields and excellent ee, as shown in Scheme 30.

**Scheme 31:**
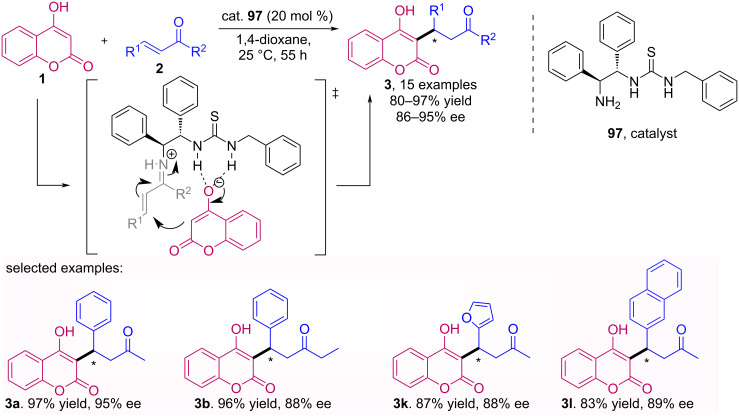
Michael addition of 4-hydroxycoumarin **1** to α,β-unsaturated ketones **2** promoted by primary amine thiourea bifunctional catalyst **97**.

An asymmetric Michael addition of 4-hydroxycoumarin (**1**) to α,β-unsaturated ketones **2** promoted by chiral primary amine thiourea bifunctional catalyst **97** was reported by Mei et al. [66]. Using the optimized conditions, a series of Michael adducts **3** were obtained in excellent yields (up to 97%) and enantioselectivities (up to 95% ee) (Scheme 31). As a highlight, optically pure (*S*)-warfarin (**3a**) was obtained in 99% ee after simple and single recrystallization.

**Scheme 32:**
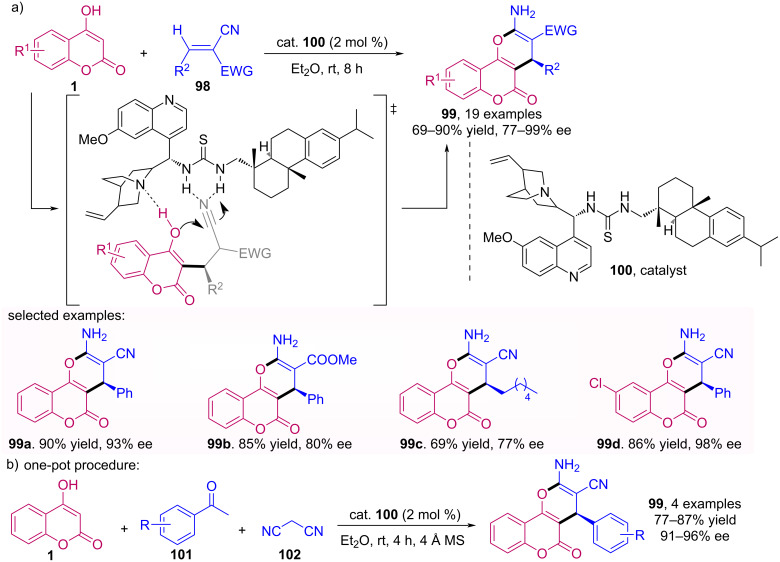
Enantioselective synthesis of functionalized pyranocoumarins **99**.

Wang’s group developed a bifunctional thiourea and abietic acid catalyst for enantioselective synthesis. In this context, they applied this catalyst in a domino reaction of pyranocoumarins **99** [67]. The procedure proved to be efficient for obtaining products with good to excellent yields and enantiomeric excesses, and in some cases starting from three components in a one-pot procedure (Scheme 32). The chiral catalyst **100** allows the addition in the least hindered *Re* face, consequently resulting in products of (*R*)-configurations, which were determined via X-ray crystallography.

**Scheme 33:**
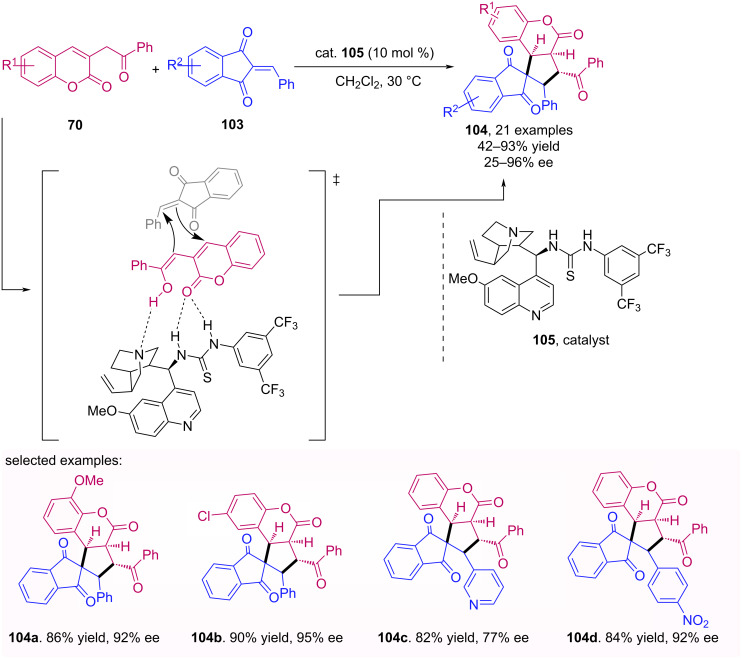
3-Homoacylcoumarin **70** as 1,3-dipole for enantioselective concerted [3 + 2] cycloaddition.

A stereoselective [3 + 2] cycloaddition with indandione alkylidenes **103** and 3-homoacylcoumarin **70** as the 1,3-dipole precursor, to generate a series of coumarin/indandione-fused spirocyclopentanes **104** bearing four contiguous stereogenic centers, was described by Chen et al. [68]. This transformation was catalyzed by a cinchona-thiourea derivative **105** furnishing the spiro compounds with good to high yield and enantioselectivity (Scheme 33). In this method two mechanisms occur in parallel, which results in the formation of the Michael adduct as a byproduct and the desired spirocyclopentanes **104**. It is noteworthy that the mechanistic studies showed that the product is formed through a concerted mechanism and therefore is not part of an intermediate adduct.

**Scheme 34:**
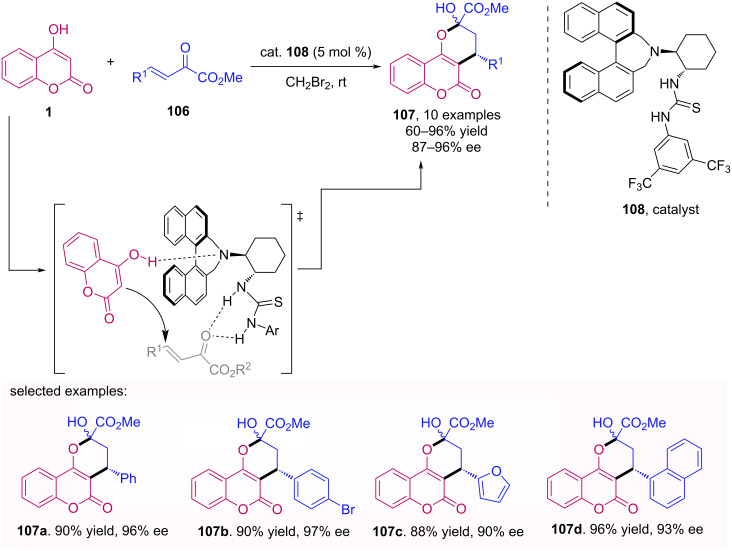
Synthesis of warfarin derivatives **107** through addition of 4-hydroxycoumarins **1** to β,γ-unsaturated α-ketoesters **106**.

A conjugate addition of 4-hydroxycoumarin (**1**) to β,γ-unsaturated α-ketoesters **106** was reported the Kim’s group [69]. In this case, a bifunctional binaphthyl-modified thiourea organocatalyst **108** was used, and among the solvents probed (such as CH_2_Cl_2_, CH_3_CN and toluene), the best results were achieved when the reaction was conducted in dibromomethane at room temperature. The use of only 5 mol % of the catalyst afforded the desired products with excellent yields and enantioselectivities (Scheme 34).

**Scheme 35:**
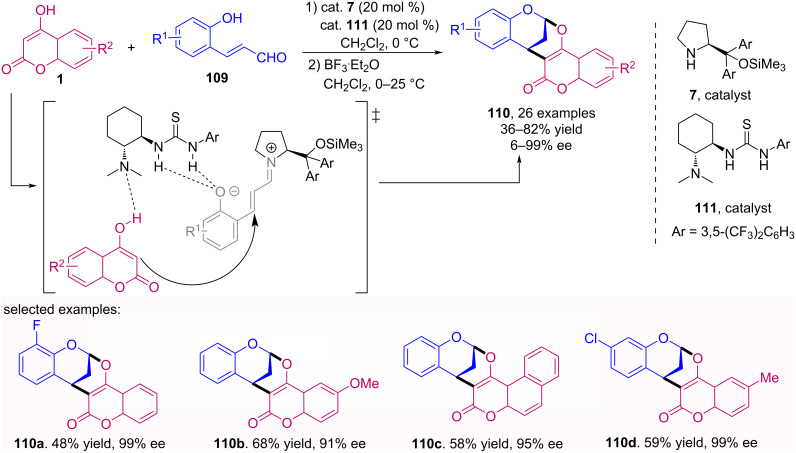
Asymmetric multicatalytic reaction sequence of 2-hydroxycinnamaldehydes **109** with 4-hydroxycoumarins **1**.

The use of multicatalytic systems have become a useful strategy for the case where it is not possible to achieve the desired transformation by using only one catalyst [70]. In this sense, an efficient asymmetric organocatalytic reaction was reported by Zhang et al. for the synthesis of 2,8-dioxabicyclo[3.3.1]nonanes [71]. A combination of catalysts **7** and **110**, involving iminium and anion-binding catalysis, respectively, has proved to be the most effective for the promotion of the conjugate addition of 4-hydroxycoumarins **1** to 2-hydroxycinnamaldehydes **109**, leading to chiral bridged bicyclic acetal products **110** with high ee (Scheme 35). The mechanistic study performed showed that possibly the phenolic hydroxy group of 2-hydroxycinnamaldehydes is important for the success of the employed catalytic system.

Finally, but not least, the phase-transfer chiral organocatalysts have also been highly explored [72–73]. Most of the PTCs are based on the skeletons of cinchona alkaloids and chiral binaphthyls, though, more recently, the strategy via introducing secondary interactions for the design of the bifunctional catalysts achieved wide application in asymmetric reactions [74].

**Scheme 36:**
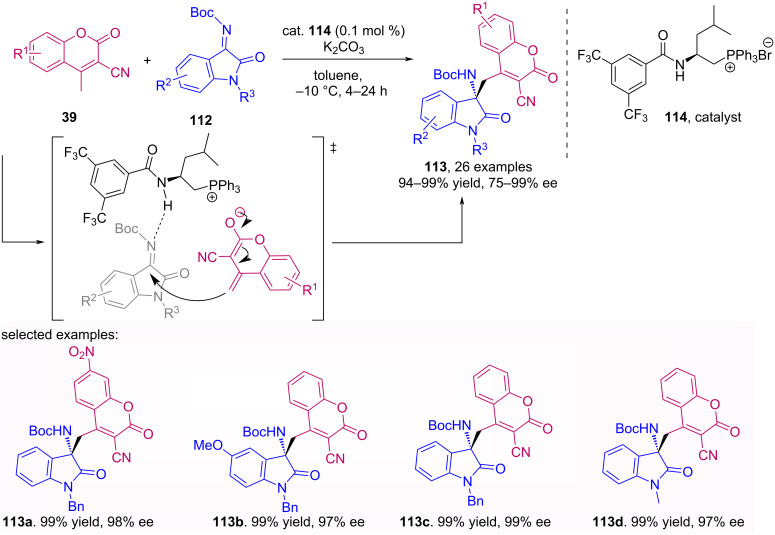
Mannich asymmetric addition of cyanocoumarins **39** to isatin imines **112** catalyzed by the amide-phosphonium salt **114**.

Wu et al. described a Mannich asymmetric addition of cyanocoumarins **39** to isatin imines **112** catalyzed by an amide-phosphonium salt **114**. This catalyst provides the formation of an ionic pair with coumarin enolate and activation of the imine by hydrogen bonding with the secondary amine, resulting in products **113** with excellent yields and high enantioselectivity [75]. This transformation draws attention because it uses only 0.1 mol % of catalyst, tolerates electron-donating and -withdrawing groups and maintains its performance in gram scale (Scheme 36).

**Scheme 37:**
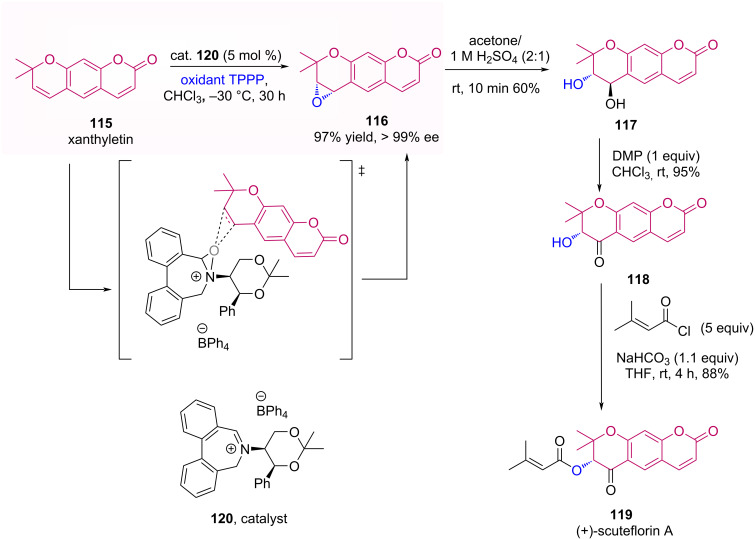
Enantioselective total synthesis of (+)-scuteflorin A (**119**).

Page et al. developed a total synthesis of the natural product (+)-scuteflorin A (**119**), being the key step an asymmetric epoxidation of xanthyletin (**115**) employing biphenylazepinium **120** as PTC together with tetraphenylphosphonium monoperoxysulfate (TPPP) as the stoichiometric oxidant [76]. The authors mentioned that this epoxidation had been previously reported using Jacoben’s (*S*,*S*)-(+)-salen-Mn(III) catalyst with 78–83% yield and 95% ee, and via organocatalysis they obtained 98% yield and ≥99% ee (Scheme 37). Furthermore, the natural product was synthesized in seven steps with 14% overall yield.

## Conclusion

Coumarin derivatives are important scaffolds for synthetic and medicinal chemistry. These structures have an interesting reactivity and can be used in diverse organic reactions, for example enantioselective organocatalyzed reactions, as presented in this review. Furthermore, coumarin derivatives are known for their wide variety of biological activities.

As can be noticed in this literature review, a wide variety of new catalysts were applied in the synthesis of coumarin derivatives and the methodologies were found to be good choices to achieve functionalized coumarins, such as the use of immobilized squaramide catalyst, which allowed the catalyst to be recycled twice with high ee. Moreover, the squaramide catalyst could also be used with low catalyst loading (1–2 mol %) providing excellent results, besides the use of only 0.1 mol % of amide-phosphonium salt for the synthesis of coumarin derivatives. Some methodologies have also proven to be highly efficient in one-pot and gram-scale procedures, which turns to be more environmentally benign.

Nevertheless, studies are still needed to accomplish procedures that allow recycling and lower catalyst loading, intertwined with the use of green solvents, in order to provide efficient and sustainable synthesis of these important pharmacologically active compounds.
